# Impaired arousal in rat pups with prenatal alcohol exposure is modulated by GABAergic mechanisms

**DOI:** 10.14814/phy2.12424

**Published:** 2015-06-09

**Authors:** Chrystelle M Sirieix, Christine M Tobia, Robert W Schneider, Robert A Darnall

**Affiliations:** 1 Department of Physiology and Neurobiology, Geisel School of Medicine at DartmouthLebanon, New Hampshire; 2 Department of Pediatrics, Geisel School of Medicine at DartmouthLebanon, New Hampshire

**Keywords:** Arousal, GABA, hypoxia, prenatal alcohol exposure

## Abstract

Prenatal alcohol exposure (PAE) increases the risk for The Sudden Infant Death Syndrome (SIDS) in human infants. In rat pups, the arousal response to hypoxia is modulated by medullary raphe GABAergic mechanisms. We hypothesized that arousal to hypoxia is impaired by PAE, and is associated with an increase in medullary GABA and enhanced GABAergic activity. Pregnant dams received an ethanol liquid diet (ETOH), an iso-caloric pair fed diet (PF) or a standard chow diet (CHOW). We first measured the time to arousal (latency), during four episodes of hypoxia in P5, P15, and P21 CHOW, PF, and ETOH pups. We also measured brainstem GABA concentration in the same groups of pups. Finally, we injected artificial cerebrospinal fluid (aCSF), nipecotic acid (NIP) or gabazine into the medullary raphe of P15 and P21 pups receiving the three diets. For statistical analysis, the PF and CHOW groups were combined into a single CONTROL group. Our main finding was that compared to CONTROL, arousal latency to hypoxia is increased in ETOH pups at P15 and P21, and the concentration of brainstem GABA is elevated at P21. NIP administration in CONTROL pups led to arousal latencies similar in magnitude to those in ETOH pups after aCSF injection. NIP injected ETOH pups had no further increases in arousal latency. We conclude that PAE impairs arousal latency and this is mediated or modulated by medullary GABAergic mechanisms.

## Introduction

The Sudden Infant Death Syndrome (SIDS) is defined as the sudden death of an infant younger than 1 year that remains unexplained after a thorough case investigation, including the performance of a complete autopsy, an examination of the death scene and a review of the infant's clinical history (Willinger et al. 1991). Several studies have indicated that maternal alcohol consumption during pregnancy is a risk factor for SIDS. Maternal drinking during the first trimester and/or during the 3 months preceding pregnancy increases the risk for SIDS by 6–8 fold (Iyasu et al. 2002). Moreover, a Danish study showed that binge drinking during pregnancy is associated with an increased risk of infant mortality, especially during the postneonatal period (Strandberg-Larsen et al. 2009). The mechanisms involved in the relationship between Prenatal Alcohol Exposure (PAE) and SIDS are not clear. In one study, maternal alcohol consumption during the first trimester was associated with sleep and arousal disturbances in infants (Scher et al. 1988), suggesting that alterations in sleep and/or arousal may contribute to the increased SIDS risk associated with PAE.

It is thought that some babies who are victims of SIDS have impaired arousal mechanisms that contribute to death (Harper and Bandler 1998; Kahn et al. 2002; Richardson and Horne 2013). Cortical arousal during both REM and non-REM sleep is incomplete in babies that eventually die of SIDS (Kato et al. 2003). Arousal from sleep is an important defense mechanism against danger-signaling stimuli, including hypoxia, hypercapnia and airway obstruction. Based on home monitor records, some SIDS infants are exposed to repeated episodes of hypoxia in the days and weeks prior to death (Kelly et al. 1991; Meny et al. 1994). Moreover, it has been shown that babies often fail to arouse in quiet sleep but always arouse in active sleep (Horne et al. 2005).

In the newborn and infant rodent, we and others have shown that repeated exposure to intermittent hypoxia causes arousal impairment characterized by a progressive lengthening of the time to arousal (latency) called “habituation” (Dauger et al. 2001; Durand et al. 2004; Darnall et al. 2010). This has also been shown in the lamb (Fewell and Konduri 1989; Johnston et al. 1998, 1999) and the piglet (Waters and Tinworth 2005). Others have shown that arousal habituation to tactile stimuli occurs in human infants (McNamara et al. 1999). However, one study indicated that human infants do not appear to habituate to hypoxia (Parslow et al. 2004).

We have also shown in rodent pups that medullary raphe GABAergic mechanisms are involved in arousal and arousal habituation (Darnall et al. 2012). Medullary raphe serotonergic and GABAergic mechanisms have both been implicated in the pathology of SIDS (Kinney et al. 2009). Indeed, abnormalities of the serotonergic system of the medulla oblongata have been found in a majority of SIDS cases (for review see (Kinney et al. 2003)). SIDS is associated with lower 5-HT and Tryptophan Hydroxylase 2 (TPH2) levels in the raphe obscurus and the lateral paragigantocellular nucleus (Duncan et al. 2010). Paterson et al. (2006) reported that SIDS cases compared to control cases have a higher number of 5-HT cells and decreased binding of 5-HT_1A_ receptors (Paterson et al. 2006).

Broadbelt et al. (2010, 2011) showed that a subset of GABAergic neurons in the medullary raphe of human infants also release 5-HT suggesting a relationship between the GABAergic and serotonergic systems during development. These authors also reported that GABA_A_ receptor binding is decreased in the medullary raphe (Broadbelt et al. 2010, 2011). Recently, we have reported that medullary GABAergic mechanisms are also involved in arousal in response to hypoxia in infant rodents. Injections into the medullary raphe of the GABAergic reuptake inhibitor, nipecotic acid (NIP), or the GABA_A_ receptor agonist, muscimol, increase the latency to arousal in response to hypoxia. In addition, injections of the GABA_A_ receptor antagonist, bicuculline, greatly attenuate arousal habituation to repeated exposures to hypoxia without changing the initial arousal latency (Darnall et al. 2012).

Prenatal alcohol exposure (PAE) has many effects on the GABAergic system in adult animals. PAE increases GABA_A_ receptor expression in the cerebral cortex of Guinea pigs and decreases the number of GABAergic neurons in the somatosensory cortex (Bailey et al. 2001, 2004). PAE also reduces the number of parvalbumin-immunoreactive GABAergic neurons in the rat cingulate cortex (Moore et al. 1998) and alters GABA_A_ receptor-mediated neurotransmission (Korpi 1994; Allan et al. 1998).

However, there is less information about the effects of PAE on the GABAergic system in the fetus and developing animal. A small number of studies have shown that mild prenatal alcohol exposure promotes premature tangential migration of primordial GABAergic interneurons into the fetal cortex (Cuzon et al. 2008) and more severe PAE increases the GABA content in the fetal brain tissue (Maier et al. 1996; Sari et al. 2010). PAE combined with early postnatal exposure also decreases the GABA level in the pons and cerebellum of three week old rat pups (Ledig et al. 1988). There is also little information on the effects of PAE on the GABAergic system specifically in the brainstem. Thus, although PAE appears to have effects on the GABAergic system in many regions of the brain, the effects of PAE on the GABAergic system in the brainstem and in particular the medullary raphe are not well understood.

Little is known about the effect of PAE on arousal or arousal habituation in response to hypoxia. Moreover, we do not know whether any PAE-induced changes in arousal latency will be associated with changes in brainstem GABA or 5-HT levels or whether manipulations of medullary GABAergic mechanisms will alter any PAE effects on arousal. In these experiments, we focused on the medullary raphe because of the association of medullary 5-HT and GABAergic dysfunction with SIDS (Kinney et al. 2009). We hypothesized that (1) PAE would delay arousal (increase arousal latency) in response to repeated episodes of hypoxia; (2) the PAE-induced increase in arousal latency would be associated with an increase in brainstem GABA levels; (3) experimentally increasing medullary raphe GABA levels by locally blocking GABA reuptake mechanisms would further increase arousal latency associated with PAE; and (4) blocking GABA_A_ receptors would decrease any PAE-associated changes in arousal habituation.

To test these hypotheses, we first determined the effect of PAE on arousal latency and habituation in response to hypoxia by exposing P5, P15, and P21 rat pups born to dams that had received alcohol during gestation to repeated episodes of hypoxia. We also determined whether PAE was associated with changes in brainstem levels of GABA and/or other neurotransmitters. We finally determined whether any arousal impairment associated with PAE was modulated by GABAergic mechanisms by locally blocking GABA reuptake sites with NIP, and GABA_A_ receptors with gabazine.

## Materials and Methods

### Ethical approval

All experimental protocols were approved by the Institutional Animal Care and Use Committee of Dartmouth College.

### Animals and feeding protocol

Sprague–Dawley rats were housed in standard cages in the Dartmouth College Center for Comparative Medicine and Research (CCMR) with standard light-cycling (7 am–7 pm). The average room temperature of the animal facility was 22°C. The offspring from first time pregnancies were used in all studies. Three liquid diets (Lieber deCarli'82; BioServ, Flemington, NJ) and a standard chow diet were provided to the dams every day around 4 pm: a low level alcohol liquid diet (12% Ethanol derived calories, 2.2% v/v), a high level (binge) alcohol liquid diet (36% Ethanol derived calories, 6.7% v/v), a control liquid diet or a standard chow diet. Water was provided ad libitum.

Our choice of ethanol concentrations was based on previous studies showing a decrease in the number of serotonergic neurons in the dorsal and median raphe and B9 neurons after daily exposure throughout gestation to a moderate concentration of an ethanol (6.6%v/v, 36% Ethanol derived calories) containing liquid diet (Druse et al. 1991; Tajuddin and Druse 1999, 2001). Other studies using a liquid diet containing a lower concentration of ethanol (4.49% v/v and 3.6% v/v) also have reported a decrease in the numbers of serotonergic neurons in the dorsal and median raphe and a reduction in the migration and development of 5-HT neurons (Zhou et al. 2001, 2005; Sari and Zhou 2004). We chose to use a “binge” paradigm alternating high and low levels of alcohol in an attempt to model human social binge drinking that might occur during early pregnancy. The timing of the exposure was based on reports that peri-conceptional alcohol exposure increased the risk for SIDS by 6–8% in a high alcohol consuming population (Iyasu et al. 2002; Kinney et al. 2003).

The dams were divided into three diet groups: A standard chow diet (CHOW group), a binge alcohol containing liquid diet consisting of 2 days of 36% Ethanol-derived calories alternating with 3 days of 12% Ethanol derived calories (ETOH group) and a control liquid diet referenced as “pair-fed” where each dam was calorically matched daily to an ETOH group dam (PF group). Starting 7 days prior to mating, dams in the ETOH group received 2 days of a control liquid diet followed by 5 days of a liquid diet containing 12% Ethanol-derived calories ad libitum. Dams in the PF group received 7 days of a control liquid diet calorically matched to an ETOH dam, and dams in the CHOW group received a standard chow diet. Mating plug detection was used to determine the first day of gestation (E0). At E0, the three groups were started on their respective diets which were continued until parturition. The ETOH dams received 2 days of the high alcohol containing liquid diet (binge period) alternating with 3 days of the low alcohol containing diet ad libitum. This binge paradigm allowed 4 binge-periods over the course of gestation. The blood alcohol concentration averaged 90–100 mg/dL during the 2-day binge period and 10–12 mg/dL during the 3 day low level exposure. This is roughly equivalent to the BAC that would be achieved in a 140 pound human adult after rapidly consuming 3–4 standard drinks (each equivalent to 0.6 fluid oz of alcohol). Each PF dam received daily administration of a liquid diet calorically matched to an ETOH dam. The CHOW dams received daily administration of a standard chow diet ad libitum. Each litter was culled to 12 pups two days after birth (P2). Overall, a total of 322 rat pups from 87 litters were included in the study.

### Corticosterone measurement in rat pups

To determine the level of stress in the pups exposed to our treatment and experimental protocols, we measured the plasma concentration of corticosterone in P19 rat pups from the ETOH and PF groups (3 litters in each group, 2 pups/litter, *n* = 6 per condition). Pups were anesthetized and the blood was drawn from the heart into a heparinized tube. The corticosterone concentration in plasma was determined using ELISA (Corticosterone ELISA kit; Enzo Life Sciences, Farmingdale, NY). The manufacturer of the kit reports that the intra-assay coefficients of variation, calculated from samples containing a low, medium, or high concentration of corticosterone, are as follow: Low concentration 8%, Medium concentration 8.4%, and High concentration 6.6% (Corticosterone ELISA kit; Enzo Life Sciences, ADI900-097). Measurements were taken at different time points of the experiments: (1) immediately after removal from the cage before starting any experiments (Baseline); (2) after pups were fitted with a vest to measure ECG and after spending about 10 min in the experimental chamber breathing room air (Chamber); and (3) after 4 bouts of hypoxia exposure (Hypoxia). Corticosterone was also measured (1) before (Presurgery); and (2) after (Surgery) surgery involving isoflurane anesthesia, placement into a stereotaxic adaptor, and microinjections.

### Arousal study

Arousal studies were done in male and female rat pups, randomly chosen from each litter. We have previously shown that both male and female pups arouse in the same manner when exposed to repeated periods of hypoxia (Darnall et al. 2010, 2012). Arousal studies were done during two time periods, two years apart. In a first study, the time from the onset of hypoxia (latency) was determined in 58 pups from 8 litters during 4 exposures to 10% oxygen. In this study, there were 12 pups in the CHOW group, 22 pups in the ETOH group, and 24 pups in the PF group. A second study was done using the same arousal protocol, but where arousal latency was determined in 31 pups from 31 litters, one pup per litter, for each of the same three groups as in the first study. In this second study there were 11 pups in the CHOW group, 12 pups in the ETOH group, and 8 pups in the PF group. In both studies arousal determination was repeated in the same animal within a day of three different ages: P5, P15, and P21. The determination of the three ages was based on several studies comparing human and rat brain growth (Dobbing and Sands 1979), neurotransmitter development (Bayer et al. 1993; Whitaker-Azmitia 2005; Maciag et al. 2006), sleep and arousal (McNamara et al. 1998; Dauger et al. 2001; Karlsson and Blumberg 2002; Durand et al. 2004; Blumberg et al. 2005; Seelke et al. 2005), and the autonomic control of heart rate (Hofer and Reiser 1969) and thermoregulation (Lagerspetz 1966; Conklin and Heggeness 1971; Mouroux et al. 1990; Anderson et al. 2003). Each age used is likely to represent different time point of the rat development in comparison to human. Thus, P5 roughly corresponds to the third trimester of gestation in the human, P15 corresponds to early infancy and the time of the highest incidence of SIDS, and P21 corresponds to early childhood.

We have previously shown that hypoxia is an effective arousal stimulus in rat pups at these ages, and our techniques have been described previously (Darnall et al. 2010, 2012). Briefly, pups were removed from the litter, and with care to keep them warm, were weighed and the sex determined. They were then fitted with a flexible vest and placed prone or on their side in a custom built cylindrical, double-walled, acrylic chamber warmed by circulating water to a thermoneutral temperature (wall °C: P5, 34; P15, 33; P21, 32). The vest held surface ECG electrodes to record heart rate (*f*_H_) and a piezoelectric film motion sensor to record respiratory frequency (*f*_R_) and body movement. A 36 gauge flexible thermocouple (Omega Engineering) was inserted into the lower colon to record body temperature (T_B_), which along with chamber wall and air temperature were measured continuously. Breathing gases were supplied from high pressure sources equipped with automated solenoid valves to allow switching between room air and 10% oxygen (premixed with balance nitrogen). Gas was heated to 30°C and humidified and directed to a 5 cc vented tube from which gas was and then drawn through the chamber under mass flow control (MFC2; Sable system, North Las Vegas, NV) at a constant 750 mL/min. The chamber volume was 310 mL for P5, 350 mL for P15 pups and 380 mL for P21 animals to assure that the decrease in chamber oxygen concentration was at the same rate for each animal. Measurements of chamber pressure (Validyne) assured that switching the gas source produced only negligible pressure change. Oxygen (Sable Systems) and carbon dioxide (CWE Inc., Ardmore, PA) analyzers continuously sampled dried gas leaving the chamber. All signals were recorded for later analysis using a commercially available data acquisition system (AD Instruments, Colorado Springs, CO).

Pups slept readily in the warmed chamber, and were judged to be awake, in quiet sleep or active sleep by observation. We and others have shown that the determination of sleep and wakefulness can be accomplished without electroencephalographic measurement in young rats and mice using behavioral criteria (Blumberg et al. 2005; Darnall et al. 2010, 2012). Wakefulness was characterized by the presence of movement and a high level of muscle tone, assessed by observation. Quiet sleep (quiet immobility) was characterized by lack of movement, low muscle tone, closed eyes, and regular *f*_H_ and *f*_R_. Active sleep was characterized by myoclonic twitching of face or limbs on a background of low muscle tone and immobility.

After an acclimation period of about 10 min in the chamber and during a period of quiet sleep, pups were exposed to 3 min of 10% oxygen followed by 4 min of normoxia and this was repeated three times. There was some variation in the normoxia period to allow the pup to achieve quiet sleep before the onset of the next trial. Thus the total cycle time (hypoxia + normoxia) for each trial was about 7 min and the total time for each experiment was approximately 40 min. The sequence of arousal in both animals and humans has been previously well described and consists of a sub-cortical activation progressing to a cortical activation resulting in a complete and behavioral arousal (Lijowska et al. 1997). Arousal from sleep after the start of the hypoxia trial was recorded by the observer and was determined by observing stereotypical forelimb and neck extension, and head rearing, a coordinated motion that likely reflects cortical activation and arousal (McNamara et al. 1998; BuSha et al. 2001; Dauger et al. 2001; Darnall et al. 2010, 2012). No pups failed to arouse to hypoxia during quiet sleep. After arousal, during the remainder of the hypoxic period, pups were active for variable periods of time, and then frequently reentered sleep. Pups were returned to the litter after each experiment. P5 pups were tattooed in order to be identified at P15 and P21.

### Measurement of medullary neurotransmitters

Six pups from each group (CHOW, PF, and ETOH) were randomly chosen from six of the previously described litters at each of the 3 ages (*n* = 54). Each pup was removed from the litter to determine the medullary levels of serotonin (5-HT), 5-Hydroxyindoleacetic acid (5-HIAA), GABA, and Glycine (GLY). To accomplish this, the pups were euthanized by a lethal intra-peritoneal injection of euthasol (pentobarbital sodium and phenytoin sodium solution, i.p.); the brains were harvested and the brainstems (medulla and pons) were dissected and rapidly frozen by immersion in cold isopentane (Sigma-Aldrich, St Louis, MO). To assess neurotransmitter content, high-pressure liquid chromatographic (HPLC) analysis was performed on each brainstem (Vanderbilt Core Facility, Vanderbilt University, Nashville, TN).

### Pharmacological study

Pups were studied from each of the three experimental groups: CHOW (15 litters), PF (11 litters) and ETOH (16 litters). One pup from each litter was studied at P15 and a different pup from the same litter was tested at P21. Nipecotic acid (NIP, 400 mmol/L, Sigma-Aldrich), gabazine (5 *μ*mol/L, Sigma-Aldrich), or artificial cerebrospinal fluid (aCSF, Na 150 mmol/L, K 3.0 mmol/L, Ca 1.4 mmol/L, Mg 0.8 mmol/L, P 1.0 mmol/L, Cl 155 mmol/L) was injected into the medullary raphe prior to arousal testing. A total of 119 pups from 42 litters were successfully injected into the target area, and were included in the final study. Our injection technique has been described previously (Darnall et al. 2012). Briefly, pups were anesthetized with isoflurane (in O_2_) (induction: 3%; maintenance 2%) and placed in a neonatal rat stereotaxic adapter. T_B_ was maintained with a heating pad. Carprofen (Rimadyl; Pfizer, New York, NY), a commonly used NSAID with no reported effect on sleep or respiration, was injected (i.m., 5 mg/kg) after induction. The skull was leveled between Bregma and Lambda, and a midline incision was made through the skin. A 1-mm diameter hole was drilled in the skull over the injection site (P15: AP -2.1 mm from Lambda, DV 10 mm from Lambda surface, P21: AP -2.3 mm from Lambda, DV -10.2 mm from Lambda surface). Microinjections targeted the midline medullary raphe encompassing portions of the Raphe Pallidus, Raphe Magnus and Raphe Obscurus. The atlas of Paxinos and Watson (2007) was used to locate the injection sites. NIP, gabazine or aCSF was administered as a single injection in each subject. Fused silica tubing (Polymicro Technologies, Lisle, IL) positioned by a micromanipulator and connected to a picospritzer delivered injectate incrementally. The fused silica tubing selected was the smallest that was rigid enough to remain straight when entering brain tissue with minimal clogging (TSP050192, inner diameter = 50 *μ*m, outer diameter = 186 *μ*m). Volume was visually measured by meniscus travel along a millimeter ruler. Pups received one 50 nL injection of either NIP, gabazine or aCSF with fluorescent latex microspheres (Lumafluor, Inc., Durham, NC) added to locate injection sites in brain tissue. The scalp was sutured and care was taken to maintain T_B_ while the animal recovered from anesthesia. Posture, locomotor activity, response to touch, *f*_H_ and T_B_ recovered after about 30 min at which point the animal was readied and the arousal experiment began.

At the conclusion of the experiment the animals were euthanized (euthasol, i.p.) and the brains were removed, rapidly frozen and stored at −80°C. Frozen brainstems were sliced (35 *μ*m) and directly collected on gelatin subbed slides. After drying, the presence of the fluorescent latex microspheres was examined to locate the center of injection, and photographs were taken. The slices were then stained with cresyl violet, more rapidly than usual to prevent fluorescent fading caused by prolonged exposures to both xylene and ethanol. A second photograph was made that included the cresyl violet staining. The photos were then merged in Photoshop (Adobe®), and an atlas (Paxinos and Watson 2007) was used as a reference for determining the injection location. Data were used only from rats where the injection site encompassed the Raphe Magnus, Raphe Pallidus or Raphe Obscurus.

### Statistical analyses

**Figure 1:**
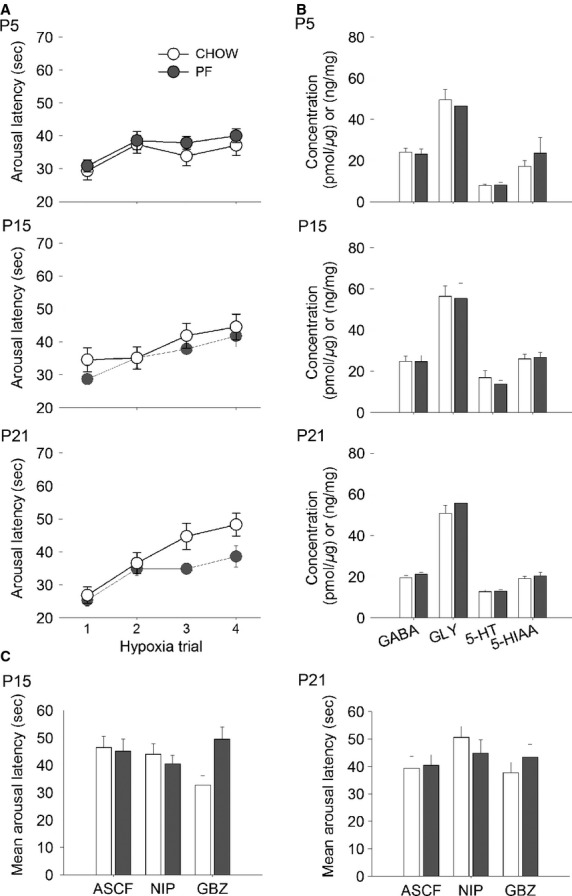
Arousal latency to hypoxia, brainstem concentration of neurotransmitters and mean arousal latency following aCSF, NIP, and gabazine injection in CHOW and PF control rat pups. (A) Arousal latency averaged across four trials of hypoxia for the PF (*n* = 32) and CHOW (*n* = 23) groups. (B) Brainstem concentrations of 5-HT, 5-HIAA, GABA and GLY for the PF (*n* = 6) and CHOW (*n* = 6) groups. (C) Mean arousal latency averaged across the four hypoxia trials following injection in the medullary raphe of aCSF, (CHOW P15 *n* = 7, P21 *n* = 6; PF P15 *n* = 7, P21 *n* = 6), NIP (CHOW P15 *n* = 7, P21 *n* = 6; PF P15 *n* = 9, P21 *n* = 6) and gabazine (CHOW P15 *n* = 7, P21 *n* = 7; PF P15 *n* = 7, P21 *n* = 5) in the PF and CHOW groups. Values are expressed as mean (SEM).

In most cases variables were compared using a MIXED model (SPSS v22) with age (P5, P15, and P21), group (CHOW, PF, and ETOH), trial (1–4), and/or injection (aCSF, NIP, gabazine), as fixed effects. For the arousal studies, age and trial were considered repeated measures and a random factor was included to account for the two studies done during two time periods. For the pharmacological study, only trial was considered a repeated measure. For the evaluation of baseline measurements taken before the first hypoxia exposure, trial was not included in the model. Kruskal–Wallis tests were used to compare neurotransmitter and corticosterone levels. For the arousal and pharmacological studies, we also found in our initial analyses that arousal latencies for the CHOW and PF groups were not different (Fig.1A and C). Similarly, for the measurement of brainstem neurotransmitter levels, values were nearly identical for the CHOW and PF groups (Fig.1B). We therefore elected to combine the PF and CHOW groups into one CONTROL group to simplify these analyses. Post hoc pairwise comparisons were corrected for multiple comparisons (Bonferroni). All data are expressed as mean (SEM).

## Results

### Baseline values

Baseline data obtained before the onset of the first hypoxia trial for the arousal and pharmacological studies are shown in Tables1 and 2. CHOW and PF data are presented both separately and combined into a single CONTROL group. For both studies, the number of males and females used are roughly equivalent. The report here describes the comparison between the ETOH and the CONTROL groups. In the arousal study (Table1), each pup was studied at three ages (P5, P15, and P21). As expected, wt increased with age across all diet groups (*P* < 0.001), and the changes in weight were not influenced by diet (group). There was a main effect of age on *f*_H_ (*P* < 0.001) such that, in general, *f*_H_ increased with age. In contrast, there was no effect of diet (group) on *f*_H_ (*P* = 0.970) at any age. There was also a main effect of age on *f*_R_ (*P* = 0.002) but no effect of group (*P* = 0.319) at any age. In the ETOH group, *f*_R_ at P5 was lower than both P15 (*P* = 0.035) and P21 (*P* = 0.042). There was no difference between ages in the CONTROL group. Although the differences in temperature are likely not clinically significant, there was a main effect of age on T_B_ (*P* < 0.001) but no effect of group (*P* = 0.201) at any age. However, in the CONTROL group, T_B_ at P21 was higher than at P5 (*P* = 0.010). In the ETOH group, T_B_ was higher at P21 than at both P5 (*P* < 0.001) and P15 (*P* = 0.021).

**Table 1:** Baseline data obtained before the first trial of the arousal study.

	P5			P15			P21		
	CHOW	PF	ETOH	CHOW	PF	ETOH	CHOW	PF	ETOH
M/F (*n*)	10/13	19/13	20/14						
wt, g	12.3 ± 0.4	12.5 ± 0.2	12.9 ± 0.5	34.4 ± 0.9	34.2 ± 0.9	37.1 ± 0.9	63.8 ± 3.5	68.9 ± 1.8	66.2 ± 2.9
*f*_H_, bpm	396 ± 9	400 ± 5	394 ± 5	440 ± 8	456 ± 5	453 ± 6	462 ± 7	470 ± 9	466 ± 9
*f*_R_, breaths/min	112 ± 17	134 ± 11	120 ± 17	141 ± 7	159 ± 12	160 ± 9	145 ± 10	139 ± 19	161 ± 10
T_B_, °C	35.4 ± 0.2	35.6 ± 0.1	35.5 ± 0.1	35.6 ± 0.1	35.8 ± 0.1	35.9 ± 0.2	35.5 ± 0.3†	36.6 ± 0.2	36.5 ± 0.2
	CONTROL			CONTROL			CONTROL		
wt, g	12.4 ± 0.2			34.7 ± 0.6			64.5 ± 1.7		
*f*_H_, bpm	395 ± 4			453 ± 4			470 ± 5		
*f*_R_, breaths/min	112 ± 8			154 ± 5			142 ± 6.9		
T_B_, °C	35.4 ± 0.1			35.7 ± 0.1			36.0 ± 0.2		

† Different between CHOW and both PF and ETOH groups (*P* < 0.001).

M/F, male/female

wt, weight

*f*_H_, heart rate

*f*_R_, respiratory rate

T_B_, body temperature.

All values are mean (SEM).

In the pharmacological study (Table2), different pups were studied at two ages, P15 and P21. As expected wt increased with age (*P* < 0.001). There was no main effect of group (*P* = 0.114), injection (*P* = 0.205) or age (*P* = 0.121) on *f*_H_. However, at P21, *f*_H_ in CONTROL pups was higher after gabazine injection compared to after both aCSF (*P* = 0.02) and NIP (*P* = 0.022) injections. There was no difference in *f*_H_ between the CONTROL and ETOH groups at any age. Similarly, there was no main effect of either group (*P* = 0.191), injection (*P* = 0.056) or age (*P* = 0.538) on *f*_R_. At P15, in the CONTROL group, NIP injection increased *f*_R_ compared to pups injected with aCSF (*P* = 0.009). At P21, CONTROL pups injected with gabazine had a higher *f*_R_ compared to aCSF (*P* = 0.016) injected pups. After NIP injection *f*_R_ was lower in the ETOH compared to CONTROL pups (*P* = 0.043). There was a main effect of group (*P* = 0.025) on T_B_ but no effect of injection (*P* = 0.720) or age (*P* = 0.310). At P21, ETOH pups injected with NIP had a higher T_B_ compared to CONTROL pups injected with NIP (*P* = 0.033).

**Table 2:** Baseline data obtained before the first trial of the pharmacology study.

	P15								
	CHOW	PF	ETOH	CHOW	PF	ETOH	CHOW	PF	ETOH
M/F (*n*)	4/3	6/1	4/2	5/2	4/5	2/4	4/3	3/4	3/5
wt, g	33.5 ± 0.8	35.0 ± 0.6	32.9 ± 0.8*						
	aCSF			NIP			Gabazine		
*f*_H_, bpm	408 ± 18	419 ± 11	440 ± 17	436 ± 18	432 ± 10	439 ± 16	440 ± 14	394 ± 17	442 ± 15
*f*_R_, breaths/min	118 ± 9	114 ± 6	124 ± 3	143 ± 8#	132 ± 5	132 ± 5	119 ± 10	124 ± 9	119 ± 3
T_B_, °C	33.1 ± 0.3	33.1 ± 0.4	33.0 ± 0.6	34.1 ± 0.4	33.4 ± 0.3	33.9 ± 0.4	33.9 ± 0.2	33.6 ± 0.7	34.4 ± 0.4
	CONTROL			CONTROL			CONTROL		
wt, g	34.3 ± 0.5								
*f*_H_, bpm	414 ± 10			434 ± 9			417 ± 12		
*f*_R_, breaths/min	116 ± 5^$$^			137 ± 4			121 ± 6		
T_B_, °C	33.1 ± 0.3			33.7 ± 0.2			33.8 ± 0.4		

	P21								
	CHOW	PF	ETOH	CHOW	PF	ETOH	CHOW	PF	ETOH
M/F (*n*)	3/3	2/4	2/5	5/1	2/4	3/3	4/3	2/3	3/3
wt, g	52.2 ± 1.2*	57.5 ± 2.1	52.0 ± 1.2*						
	aCSF			NIP			Gabazine		
*f*_H_, bpm	428 ± 21	421 ± 17	445 ± 17	419 ± 17	432 ± 25	436 ± 14	463 ± 7	480 ± 10	459 ± 11
*f*_R_, breaths/min	115 ± 9	123 ± 6	121 ± 4	140 ± 12	135 ± 6	118 ± 7$	147 ± 11##	131 ± 6.8	127 ± 5.6
T_B_, °C	33.7 ± 0.8	33.4 ± 2.0	35.3 ± 0.5	34.0 ± 0.7*	31.1 ± 2.3	34.8 ± 0.5$, **	33.7 ± 0.7	33.5 ± 0.4	34.5 ± 0.5
	CONTROL			CONTROL			CONTROL		
wt, g	54.7 ± 1.2								
*f*_H_, bpm	425 ± 13			425 ± 14			470 ± 6&,$		
*f*_R_, breaths/min	118 ± 5			138 ± 7			141 ± 7&		
T_B_, °C	33.6 ± 1.0			32.5 ± 1.2			33.7 ± 0.4		

The weight (wt) was measured at P15 and P21 for all CHOW, PF, ETOH and CONTROL pups independent of injections: ^*^ different from PF (*P* < 0.05). All three diets and the CONTROL group are compared for *f*_H_, *f*_R_ and T_B_:

* different from PF NIP (^*^P < 0.05, ^*^^*^P < 0.01)

# different from CHOW aCSF (^#^*P* < 0.05, ^##^*P* < 0.01);

$ different from CONTROL NIP (^$^*P* < 0.05, ^$$^*P* < 0.01) and

& different from CONTROL aCSF (^&^*P* < 0.05).

M/F, male/female

wt, weight

*f*_H_, heart rate

*f*_R_, respiratory rate

T_B_, body temperature.

All values are mean (SEM).

### Corticosterone levels

**Figure 2:**
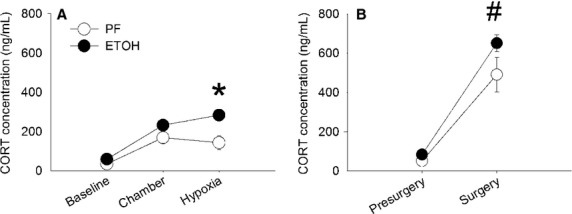
Plasma corticosterone concentrations in PF (*n* = 6) and ETOH (*n* = 6) pups at P19. (A) Plasma corticosterone concentration for three conditions: Baseline, Chamber, and Hypoxia. (B) Plasma corticosterone concentrations for two conditions: Presurgery and Surgery. *Significant difference between ETOH and PF groups for Hypoxia condition and between Hypoxia and Baseline in ETOH pups (*P* < 0.05). ^#^Significant difference between Surgery and both Baseline and Presurgery for PF and ETOH pups (*P* < 0.01). All values are expressed as mean (SEM).

Figure2A shows the plasma corticosterone levels for the PF and ETOH groups measured at P19 for the three conditions studied: Baseline, Chamber, and Hypoxia. Figure2B shows the corticosterone level from the two conditions Presurgery and Surgery. Immediately after removal from the litter and before any significant handling, Baseline mean corticosterone levels in the ETOH pups were not significantly different than those in the PF pups. After spending 10 min in the chamber (Chamber), and before the first exposure to hypoxia, mean corticosterone levels increased in both the PF and ETOH groups but there remained no difference between levels in the PF and ETOH groups. Interestingly, corticosterone levels did not significantly increase further in either group after four 3 min repeated exposures to 10% oxygen (Hypoxia). However, at the end of the four hypoxia trials, the mean corticosterone level in the ETOH group was higher than in the PF group (*P* = 0.009) and higher than the Baseline level (*P* = 0.024). The Presurgery levels of corticosterone were also not different between the two groups and not different from the Baseline values. During anesthesia and stereotaxic surgery, corticosterone levels increased dramatically in both the ETOH (*P* = 0.002) and PF (*P* = 0.007) groups compared to Presurgery condition, but the increases were not different between the two groups.

### Arousal latency

**Figure 3:**
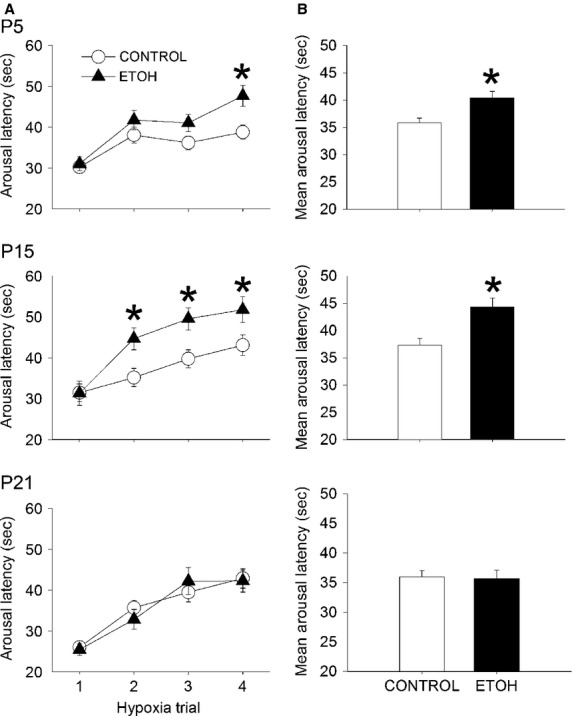
Arousal latencies in response to hypoxia at 3 different ages P5, P15, and P21. Each pup was tested at all three ages. (A) Arousal latency averaged across the four hypoxia trials in ETOH (*n* = 34) and CONTROL (*n* = 55) pups at P5, P15, and P21. At all three ages, the arousal latency progressively increases, reflecting habituation. (B) Mean arousal latency averaged across trials for ETOH and CONTROL pups at P5, P15, and P21. *Significant difference from CONTROL (*P* < 0.05). All values are expressed as mean (SEM).

Arousal latencies for the three ages studied and across the four trials of hypoxia are shown in Fig.3A. There were main effects of group (*P* = 0.009), age (*P* = 0.001), trial (*P* < 0.001) and an interaction between group and age (*P* = 0.016). The mean arousal latencies, averaged across trials, shown in Fig. 3B were significantly longer in the ETOH group compared to the CONTROL group at both P5 (ETOH = 40.37 ± 1.21 sec; CONTROL = 35.80 ± 0.89 sec, *P* = 0.019) and P15 (ETOH = 44.31 ± 1.61 sec; CONTROL = 37.32 ± 1.17 sec, *P* = 0.001). At P5, the arousal latency was longer in the ETOH group during trial 4 (*P* = 0.004), whereas at P15, latencies in the ETOH group were significantly longer during trial 2 (*P* = 0.006), trial 3 (*P* = 0.002) and trial 4 (*P* = 0.013). However, there was no difference in arousal latency between groups at P21. The progressive increase in arousal latency across trials (a measure of habituation) was estimated by calculating the slope (s/trial). There was a progressive increase in arousal latency across trials in both groups and at all ages, but no difference among groups or ages.

### Brainstem neurotransmitter concentrations

**Figure 4:**
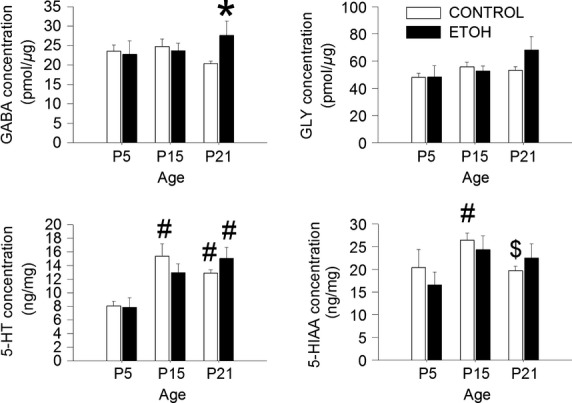
Concentrations of GABA, GLY, 5-HT, and 5-HIAA from the brainstems of CONTROL (*n* = 12) and ETOH (*n* = 6) groups at P5, P15, and P21.*significant difference from CONTROL (*P* < 0.05), ^#^Significant difference from P5 (*P* < 0.01), ^$^Significant difference from P15 (*P* < 0.05). All values are expressed as mean (SEM).

The concentrations of 5-HT, 5-HIAA, GABA and GLY were measured in the brainstems of PF, CHOW and ETOH pups at P5, P15 and P21. The PF and CHOW groups were combined into a single CONTROL group for analysis (see Fig.1). Figure4 shows the concentrations of the amino acids (GABA and GLYCINE) and monoamines (5-HT and 5-HIAA) by group and age. The concentrations of GLY, 5-HT or 5-HIAA were not different between the ETOH and CONTROL groups at any age. Similarly, the GABA concentrations at P5 and P15 were not significantly different between the ETOH and CONTROL groups. However, at P21, the brainstem GABA concentration in the ETOH group was significantly higher than in the CONTROL group (ETOH = 27.60 ± 3.62 pmol/*μ*g; CONTROL = 20.29 ± 0.68 pmol/*μ*g, *P* = 0.018). Neurotransmitter levels were influenced by age. In the CONTROL group, the concentration of 5-HT was lower at P5 compared to both P15 and P21 (*P* < 0.001) and the concentration of 5-HIAA was lower at P15 (*P* = 0.012) compared to both P5 (*P* = 0.012) and P21 (*P* = 0.042). In the ETOH group, the concentration of 5-HT was lower at P5 compared to P21 (*P* = 0.007). However, there were no differences in the GABA and GLY levels across ages.

### Pharmacological study

#### Injection locations

**Figure 5:**
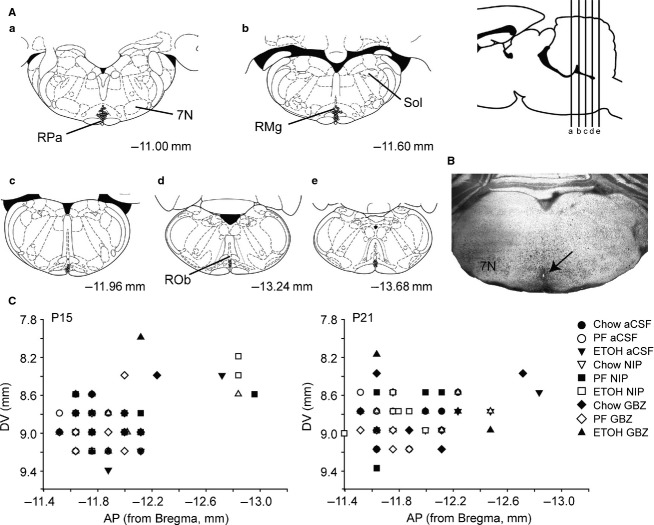
Locations of drug injections (aCSF, NIP, and gabazine) from all studied pups (ETOH, PF and CHOW) at P15 and P21 (*n* = 119). (A) Schematic showing the locations of microinjections according to the Paxinos and Watson atlas (2007). The sagittal view represents the level of frontal sections (a, b, c, d, and e). Each grey dot corresponds to an injection located in the medullary raphe (either Raphe Magnus, Raphe Obscurus or Raphe Pallidus). Only well-targeted injections are drawn. (B) An example of a section counterstained with cresyl violet showing the presence of fluorescent beads at the location of injection within the medullary raphe. (C) Histogram of the Dorsoventral (DV) coordinate as a function of the Antero–posterior (AP) coordinate of each well-targeted injection at both P15 and P21.

Injection locations of the study drugs were determined by identifying the location of the fluorescent micro-beads. We determined that the injection sites had an oval shape with a rostrocaudal dimension of ∽400 *μ*m and a lateral dimension of ∽215 *μ*m. Of the 170 rat pups injected, beads were located within the Raphe Magnus, Raphe Pallidus or Raphe Obscurus in 119. Most injections were located in the rostral part of the medullary raphe encompassing the Raphe Magnus and the Raphe Pallidus, between −11.00 mm and −11.60 mm with reference to the Bregma (Paxinos and Watson 2007). Thus, 119 pups were included in the analyses. Figure5 shows the location of drug injections (aCSF, NIP and gabazine) for all 119 cases studied at both P15 and P21. The remaining rats with an injection located outside the target area were excluded from the final analysis. A total of 64 pups were included at P15 and 55 were included at P21.

#### Arousal test

Arousal latencies were evaluated at P15 and P21 across the four hypoxia trials (trial) in CONTROL and ETOH pups (group) for each drug tested: aCSF, NIP, and gabazine (injection). There was a main effect of age (*P* = 0.001) and an interaction between group and age (*P* < 0.001). We therefore performed separate analyses for each age. To further simplify the analyses, we compared the effects of each drug (NIP or gabazine) to aCSF. Thus, a mixed model for each age was performed comparing the effect of NIP and aCSF, and gabazine and aCSF. At P15, there were no main effects of group (*P* = 0.133), or injection (*P* = 0.783) but there was a main effect of trial (*P* < 0.001) for each analysis. In contrast, at P21, the comparison of the aCSF and NIP injections showed a main effect of both group (*P* = 0.007) and injection (*P* = 0.031), as well as for trial (*P* < 0.001). However, the comparison between aCSF and gabazine injections showed only main effects for group (*P* = 0.002) and trial (*P* < 0.001) but not for injection (*P* = 0.816).

**Figure 6:**
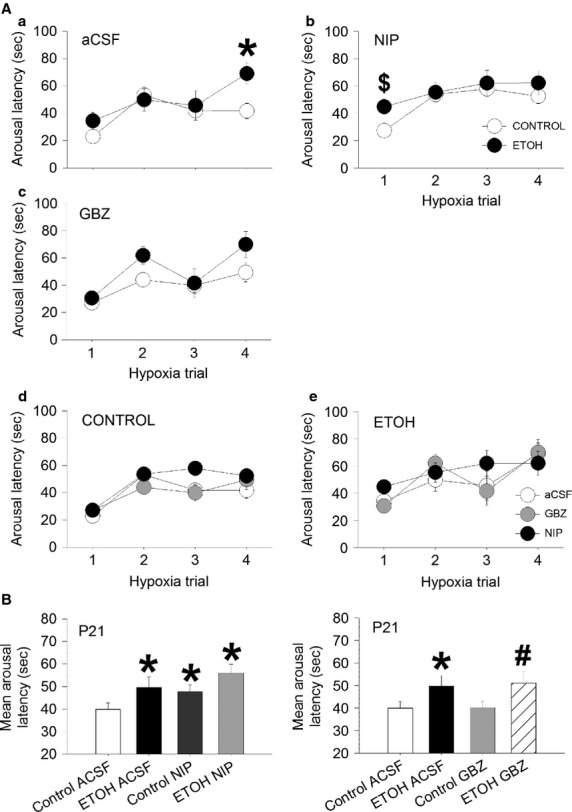
Arousal latencies in response to hypoxia in P21 rat pups from ETOH or CONTROL groups after injection of either aCSF (ETOH *n* = 7, CONTROL *n* = 12), NIP (ETOH *n* = 6, CONTROL *n* = 12) or gabazine (ETOH *n* = 6, CONTROL *n* = 12), in the medullary raphe. (A) Arousal latency across trials for the ETOH and CONTROL pups, a) injected with aCSF. b) injected with NIP, c) injected with gabazine, d) Arousal latency across trials for the three injections, aCSF, NIP, and gabazine in the CONTROL pups, e) Arousal latency across trials for the three injections, aCSF, NIP, and gabazine in the ETOH pups. (B) Mean arousal latency averaged across trials at P21 for both ETOH and CONTROL pups injected with either, aCSF, NIP or gabazine. *Significant difference from CONTROL/aCSF (*P* < 0.05), ^#^Significant difference from CONTROL/gabazine, ^$^Significant difference from CONTROL/NIP. All values are expressed as mean (SEM).

Figure6 shows the arousal latency across the four trials of hypoxia and also averaged across trials in P21 CONTROL and ETOH rat pups injected with aCSF, NIP, and gabazine. In the 21 day old pups, the effect of NIP on the mean arousal latency of pups prenatally exposed to a control diet was roughly the same magnitude as the effect of prenatal exposure to the ETOH diet after injection with aCSF. Thus, the mean arousal latency of the ETOH pups injected with aCSF (49.67 ± 4.61 sec) was significantly greater than the latency in the CONTROL pups injected with aCSF (39.90 ± 2.87 sec, *P* = 0.035). Similarly, the mean arousal latency of the CONTROL pups injected with NIP (47.77 ± 3.12 sec) was significantly greater than the latency in the CONTROL pups injected with aCSF (*P* = 0.044). Moreover, the mean arousal latency of the ETOH pups injected with NIP (56.01 ± 3.97 sec) was not significantly greater than the latency of CONTROL pups injected with NIP (47.77 ± 3.12 sec, *P* = 0.084).

In contrast, compared to injection of aCSF, the injection of gabazine into the medullary raphe did not significantly change arousal latency in pups either prenatally exposed to an ETOH or a CONTROL diet. However, The mean arousal latency in pups prenatally exposed to ETOH and injected with gabazine (51.03 ± 5.04 sec) was greater than in pups exposed to a CONTROL diet injected with gabazine (40.09 ± 2.95 sec, *P* = 0.025). The progressive increase in arousal latency across hypoxia trials was also assessed by measuring the slope across trials. There was no main effect of group or injection at either P15 or P21 in any of the analysis indicating that neither NIP nor gabazine had any effect on arousal habituation.

## Discussion

### Arousal is impaired by prenatal alcohol exposure

Our major finding is that in our rodent model, prenatal exposure to a binge alcohol paradigm impairs arousal in response to repeated exposures to hypoxia. Thus, pups prenatally exposed to alcohol had longer arousal latencies in response to hypoxia compared to pups prenatally exposed to a control diet. To the best of our knowledge, these are the first experiments to establish a link between prenatal alcohol exposure (PAE) and an impaired arousal response to hypoxia in young rat pups. Arousal is an important protective mechanism in response to potentially harmful stimuli and failure to arouse after several consecutive exposures to hypoxia may contribute to the pathogenesis of SIDS (Harper and Bandler 1998; Kahn et al. 2002; Richardson and Horne 2013). Moreover, it has also been suggested that infants may habituate to repeated hypoxic events and thus fail to respond after several stimulations (Horne et al. 2005). We and others have confirmed that arousal habituation does occur in rodent pups in response to repeated exposures to hypoxia (Dauger et al. 2001; Durand et al. 2004; Darnall et al. 2010). In this report, we show that PAE increases the mean time to arousal across four exposures to hypoxia in rat pups at an age corresponding to the critical age for SIDS in human infant. However, we did not find an effect of PAE on arousal habituation.

The effect of PAE on arousal latency was dependent on age in both the arousal and pharmacological studies, however, the age of maximum effect was different in the two experiments; the effects started at P5 but were the greatest at P15 in the arousal experiments whereas they were apparent only at P21 in the pharmacological study. The reason for this discrepancy is not clear. One possibility is that in the nonpharmacological arousal studies each pup was studied at all three ages. Thus, not only were they “acutely” exposed to four bouts of hypoxia, but they were also “chronically” exposed at three different ages. In contrast, in the pharmacological study, different pups were studied at each age (P15 and P21). It is possible that the repeated exposures at P5-induced sensitization, enhancing the effects of PAE at P15. In the piglet, acute exposures to intermittent hypercapnic hypoxia increased arousal latency and this was exacerbated after 4 days (chronic) of repeated exposures (Waters and Tinworth 2005). In addition, the pharmacological experiments involved a brief period (40 min) of isoflurane anesthesia and surgery. It is possible that, even after assuring that the recovery of heart rate, motor activity, and body temperature was complete, developmentally related effects on the depth and recovery from anesthesia may have influenced the age of the maximal effect of PAE on arousal. However, our previous findings showed that arousal latencies after anesthesia and surgery are comparable to the arousal latencies measured in pups that had no anesthesia or surgery. This suggests that the effect of anesthesia or surgery, at least in our experimental paradigm, is negligible on arousal (Darnall et al. 2012). Other investigators have also shown that isoflurane anesthesia does not affect postanesthesia sleep architecture (Jang et al. 2010). It is therefore less likely that our differences reflect an anesthesia/surgery effect.

### PAE-related arousal impairment is mediated or modulated by brainstem GABAergic mechanisms

We had postulated that (1) arousal impairment associated with PAE would be accompanied by increases in brainstem GABA; (2) arousal latencies would be further lengthened by inhibiting GABA reuptake; and (3) any increases in arousal habituation would be reduced by blocking GABA_A_ receptors. We confirmed that PAE is associated with an increase in the concentration of brainstem GABA at P21. Our pharmacological experiments showed that at the same age the increase in arousal latency in CONTROL pups produced by NIP injection into the medullary raphe was of the same magnitude as the increase in latency produced by PAE alone (ETOH group). We failed, however, to demonstrate a reduction in arousal latency with gabazine injection.

Although others have reported changes in the GABAergic system in several regions of the brain after PAE, our present work is the first to consider the effect of PAE on medullary GABAergic mechanisms. We previously demonstrated that arousal latency and habituation in response to hypoxia are mediated or modulated by medullary GABAergic mechanisms. Microinjections of both nipecotic acid (NIP) and muscimol directly into the midline medullary raphe increased arousal latency whereas bicuculline eliminated habituation (Darnall et al. 2012). In the present work, we did not study the effects of muscimol, because it provides no specific information about endogenous GABA. Instead, we elected to use NIP to elevate extracellular GABA level in the caudal raphe. NIP has a high affinity for the GABA transporters GAT1 and GAT3 and blocks the reuptake of GABA from the synaptic cleft into both neurons and glia to increase inhibitory activity (Schousboe et al. 1979; Borden et al. 1994).

Taken together, our findings suggest that the increase in arousal latency associated with PAE is mediated or modulated by medullary raphe GABAergic mechanisms, perhaps by increasing the concentration of ambient GABA. Our data do not allow us to determine the origin of the increase in medullary raphe GABA. It has been estimated that approximately 45% of all synapses in the Raphe Magnus are GABAergic (Cho and Basbaum 1991). GABA is also co-expressed with other neurotransmitters within the Raphe Magnus, including 5-HT (Millhorn et al. 1988; Stamp and Semba 1995; Chen et al. 2013) and Glycine (Hossaini et al. 2012). Also, Chen et al. (2013) showed that GABA is expressed by Neurokinin-1 expressing neurons (Chen et al. 2013). Thus, the increase in medullary GABA after PAE may originate from local GABAergic interneurons, neurons of other phenotypes in the medullary raphe that also synthesize GABA, or from GABAergic neurons projecting to this area.

Interestingly, the microinjection of NIP in pups prenatally exposed to ETOH did not further increase arousal latency above that in pups exposed prenatally to a control diet injected with NIP or in pups prenatally exposed to ETOH injected with aCSF. Since our data show that PAE is associated with increases in brainstem GABA, and since NIP prevents GABA re-uptake, increasing the level of ambient GABA, it is possible the combined effects might saturate postsynaptic GABAergic receptors resulting in no further effect of NIP on arousal after PAE. The result would be no additive effect NIP and PAE on arousal latency. Future experiments would aim to determine whether arousal latency is dependent on GABA release or receptor saturation mechanisms.

Gabazine did not have any effect on arousal latencies in the pups prenatally exposed to ETOH suggesting that the effect of PAE on arousal latency is not mediated through GABA_A_ receptors. Thus, the arousal latencies remained increased in the ETOH pups despite the gabazine injection. Our previous study showed that bicuculline, another GABA_A_ receptor antagonist, also did not reduce the mean arousal latency averaged across four trials of hypoxia, but it did effectively eliminate arousal habituation (Darnall et al. 2012). This led us to hypothesize that the phenomenon of habituation depends on GABA_A_ receptor activation. In the current study, however, we also did not observe any effect of gabazine on habituation in the pups prenatally exposed to a control diet injected with aCSF. The reason for the apparent differences in the actions of the two GABA_A_ receptor antagonists in the CONTROL animals is unclear. It is possible that the effects of bicuculline we observed in our previous study were not due to blocking GABA_A_ receptors but to some other mechanism. There is some evidence that bicuculline may have other effects, at least in the sensory cortex, not related to GABA_A_ receptor blockade (Kurt et al. 2006), that involve calcium-dependent potassium channels (Johansson et al. 2001). Alternatively, it is possible that the dose of gabazine we used was too low, or that the half-life of the effect after the single injection was too short resulting in only partial or ineffective GABA_A_ receptor blockade. This would have resulted of a lack of effect both on habituation in the CONTROL animals and on mean arousal latency in the ETOH pups. We did not test this possibility by using larger doses. Thus, it remains unclear whether the effect of PAE on arousal is due to GABA_A_ receptor activation. Future experiments, perhaps using several doses of both gabazine and bicuculline, would need to be performed to answer this question.

Another difference between gabazine and bicuculline is their effect on synaptic and extrasynaptic GABAergic receptors. Bicuculline is effective on both types of receptors whereas gabazine acts primarily on synaptic receptors (Bai et al. 2001). Moreover, studies have shown that alcohol potentiates extrasynaptic GABA_A_ receptors in the cerebellar cortex (for review (Valenzuela and Jotty 2015)) and PAE increases *δ*-subunit expression, only found in extrasynaptic receptors, of cerebellar granule neurons in rats (Diaz et al. 2014). Also, PAE increases GABA release in the cortex (Maier et al. 1996; Cuzon et al. 2008; Sari et al. 2010), and we also show this increase in the brainstem. Our data show that an elevation of ambient GABA level by NIP injections or by PAE increases arousal latency. Altogether, this suggests that arousal latency to hypoxia may not be mainly regulated by synaptic but rather by extrasynaptic GABA_A_ mechanisms.

### Limitations

One of the limitations of studies involving prenatal exposure to alcohol is the choice of a control group. It remains unclear whether there is an adequate control group with which to compare effects, behavioral or otherwise, of prenatal exposure to alcohol. We used a statistical strategy to combine the PF and CHOW group into a single CONTROL group. Nevertheless, it is clear that PF and CHOW fed pregnant dams are not receiving equivalent diets. Dams in the PF group are fed a liquid diet matching the calories taken by the ETOH group. Overall, ETOH fed dams take in fewer calories than if they had been fed a chow diet ad libitum, thus the PF dams also receive fewer calories and are constantly hungry. Other investigators have suggested that a PF group may be another treatment group, rather than a true control (Weinberg, 1984). The CHOW control group receives more calories, but is not a liquid diet, and therefore also not a true control. We did not include a liquid diet with unlimited caloric intake in the current study.

Our corticosterone measurements suggest that PAE enhances the stress response in pups similar to what has been observed in adults exposed to chronic mild stress (Hellemans et al. 2008), but that hypoxia exposure itself does not substantially increase the level of stress over and above handling and separation from the dam and siblings. It has been shown that a high dose of corticosterone, administered to rats, increases wakefulness and decreases time spent in NREM sleep (Bradbury et al. 1998; Vázquez-Palacios et al. 2001). Consequently, we would have anticipated that the enhanced stress response in the PAE pups would have shortened, rather than lengthened the time to arousal in response to hypoxia. It is therefore reasonable to conclude that the PAE-related increase in the stress response does not explain our findings. Indeed, it is possible that the increase in arousal latency that we observed in the pups after prenatal exposure to alcohol might have even been greater in the absence of the increased stress response.

In our pharmacological study, we used the center of the injection of our fluorescent beads to determine whether the injection was in the medullary raphe. The dispersal distances from the center of the injection for the drugs we used (aCSF, NIP or gabazine) are unknown and are likely to be different from the spread of the fluorescent beads. There is a chance that injected drugs may have reached regions outside of the medullary raphe. Future experiments could more specifically target the other nuclei such as the lateral paragigantocellular nucleus (LPGi) or the ventral (GiV) and alpha (GiA) parts of the gigantocellular nucleus.

### Summary

In summary, to the best of our knowledge, these are the first experiments that demonstrate that arousal latency to successive bouts of hypoxia is impaired in rat pups prenatally exposed to alcohol. We also showed that PAE is accompanied by an increase of the level of brainstem GABA. In addition, GABA reuptake blockade in pups receiving a control diet, presumably by increasing ambient GABA, increased arousal latency to roughly the same level as PAE alone. Altogether, our data suggest that the increase in arousal latency in PAE rats is modulated or mediated by GABAergic mechanisms.

It is currently accepted that alcohol consumption during pregnancy increases the risk of poor neurodevelopmental outcome (Riley and McGee 2005) and several studies suggest that PAE increases the risk for SIDS (Iyasu et al. 2002; Strandberg-Larsen et al. 2009). Arousal impairment continues to be hypothesized as an important contributor to SIDS (Harper and Bandler 1998; Kahn et al. 2002; Kinney et al. 2009; Richardson and Horne 2013) and our findings link maternal ethanol drinking and arousal impairment.

In addition to the serotonergic deficiencies reported to occur in a large subset of SIDS infants (Paterson et al. 2006), abnormalities in GABA_A_ receptor binding have been demonstrated in these infants suggesting a role for the GABAergic system (Broadbelt et al. 2011). Our current data suggest a role of the GABAergic system on the effect of PAE on arousal impairment. Others have reported an effect of PAE on the number of 5-HT neurons (Tajuddin and Druse 1999; Sari and Zhou 2004) but whether or not PAE results in a change in the number of GABAergic neurons is unknown. The increase in GABA levels that we observed suggests that either there may be an increased number of GABAergic neurons or there is an increase in GABAergic neuronal activation as a result of PAE. Future studies will determine the number of both medullary GABAergic and serotonergic neurons in rat pups prenatally exposed to alcohol.

## Supporting Information

**Figure S1**. Excel worksheet showing plasma corticosterone levels measured in PF and ETOH rats during five conditions: Baseline, Chamber, Hypoxia, Presurgery, and Surgery. The data contributed to Fig. 2.

**Figure S2.** Excel worksheet showing the arousal latency for each CONTROL and ETOH rat at P5, P15, and P21 for each of the four trials. The data contributed to Fig. 3.

**Figure S3**. Excel worksheet showing the concentration of GABA, GLY, 5-HT, and 5-HIAA at P5, P15, and P21 of CONTROL and ETOH rats. The data contributed to Fig. 4.

**Figure S4**. Excel worksheet showing the arousal latency of CONTROL and ETOH rats at P15 and P21, injected with either aCSF, gabazine or NIP at each four trials. The data contributed to Fig. 6.
